# Examining the Effect of Work‐Life Imbalance on Cognitive Complaints

**DOI:** 10.1002/alz.093553

**Published:** 2025-01-09

**Authors:** Rachel McCray, Daisha Oruru, Clarence Locklear, Omonigho M Bubu, Arlener Turner

**Affiliations:** ^1^ University of Miami, Atlanta, GA USA; ^2^ University of Miami, Miami, FL USA; ^3^ NYU Grossman School of Medicine, New York, NY USA

## Abstract

**Background:**

Subjective cognitive complaints (SCCs) are self‐reported cognitive concerns, often without objective impairment and are associated with higher risk of MCI and/or dementia. Moreover, the related subjective cognitive decline is suggested as a form of cognitive impairment, and precursor to Alzheimer’s disease and related dementias (ADRD). Sleep, stress, and anxiety have been shown to explain significant portions of variance in relationships between multi‐morbidity and SCCs. Leisure/social time can mediate stress and negative moods which may prevent worsening cardiovascular and cognitive decline. While there are many barriers to leisure, the most universal limitation is lack of time. Specifically the US has less vacation days and more expected work hours than other nations, leaving little time for rest, resulting in a work‐life imbalance (WLI) causing added stress and possible mood disorders. Though discussions are rising around federal minimum wage, and remote work, there’s little insight into WLI’s impact on long term health outcomes and cognition.

**Method:**

We explored the effect of markers of WLI (ex: stress, sleep, life satisfaction) on the presence of SCCs in participants (N = 89,976, 51.6% Female, 16.0% Black, M_age_ = 35.64 years) from the 2010 National Health Interview Survey. WLI was defined via a composite score of the subjective measures work‐family responsibility compatibility, social satisfaction, and hours slept. SCC was defined via self‐report of difficulty remembering or concentrating, with reporting at least some difficulty representing the presence of a SCC.

**Result:**

There was a significant association between WLI and greater reporting of SCCs; (X^2^_2,_ N = 379) = 7.145, p = 0.028. ANOVA analysis further demonstrated the significant influence of a greater presence of WLI on SCCs; F(2,376) = 10.29, p<.001. Those who experienced a higher frequency of SCCs (e.g. difficulty remembering things “all of the time”; [M = 2.4231]) scored highest on the WLI scale followed by the next highest “frequently” (M = 2.3456) and then “sometimes” (M = 2.0428).

**Conclusion:**

Our results demonstrate potential harmful effects of WLI on the presence SCCs. These results confirm previous research connecting stress and other modifiable risk factors for AD like impaired sleep to SCCs, suggesting an avenue for intervention. Further research is needed to fully illuminate how this relation applies to cognitive decline and ADRD.

